# QTL Mapping and Data Mining to Identify Genes Associated With the *Sinorhizobium fredii* HH103 T3SS Effector NopD in Soybean

**DOI:** 10.3389/fpls.2020.00453

**Published:** 2020-05-19

**Authors:** Jinhui Wang, Jieqi Wang, Chao Ma, Ziqi Zhou, Decheng Yang, Junzan Zheng, Qi Wang, Huiwen Li, Hongyang Zhou, Zhijun Sun, Hanxi Liu, Jianyi Li, Lin Chen, Qinglin Kang, Zhaoming Qi, Hongwei Jiang, Rongsheng Zhu, Xiaoxia Wu, Chunyan Liu, Qingshan Chen, Dawei Xin

**Affiliations:** ^1^Key Laboratory of Soybean Biology of Chinese Ministry of Education, Key Laboratory of Soybean Biology and Breeding/Genetics of Chinese Agriculture Ministry, College of Agriculture, Northeast Agricultural University, Harbin, China; ^2^Jilin Academy of Agricultural Sciences, Changchun, China

**Keywords:** symbiosis, T3SS effector, NopD, quantitative trait locus (QTL), haplotype, soybean

## Abstract

In some legume–rhizobium symbioses, host specificity is influenced by rhizobial type III effectors-nodulation outer proteins (Nops). However, the genes encoding host proteins that interact with Nops remain unknown. In this study, we aimed to identify candidate soybean genes associated with NopD, one of the type III effectors of *Sinorhizobium fredii* HH103. The results showed that the expression pattern of NopD was analyzed in rhizobia induced by genistein. We also found NopD can be induced by TtsI, and NopD as a toxic effector can induce tobacco leaf death. In 10 soybean germplasms, NopD played a positively effect on nodule number (NN) and nodule dry weight (NDW) in nine germplasms, but not in Kenjian28. Significant phenotype of NN and NDW were identified between Dongnong594 and Charleston, Suinong14 and ZYD00006, respectively. To map the quantitative trait locus (QTL) associated with NopD, a recombinant inbred line (RIL) population derived from the cross between Dongnong594 and Charleston, and chromosome segment substitution lines (CSSLs) derived from Suinong14 and ZYD00006 were used. Two overlapping conditional QTL associated with NopD on chromosome 19 were identified. Two candidate genes were identified in the confident region of QTL, we found that NopD could influence the expression of *Glyma.19g068600* (FBD/LRR) and expression of *Glyma.19g069200* (PP2C) after HH103 infection. Haplotype analysis showed that different types of *Glyma.19g069200* haplotypes could cause significant nodule phenotypic differences, but *Glyma.19g068600* (FBD/LRR) was not. These results suggest that NopD promotes *S. fredii* HH103 infection *via* directly or indirectly regulating *Glyma.19g068600* and *Glyma.19g069200* expression during the establishment of symbiosis between rhizobia and soybean plants.

## Introduction

Soybean [*Glycine max* (L.) Merr.] is a widely grown commercial crop around the world and supplies a large amount of protein and oil for humans and animals (Xin et al., 2016). Nitrogen is an indispensable element for soybean growth and an important limiting factor in crop production. Nowadays, huge amounts of nitrogen fertilizers are applied to improve crop production, but nitrogen fertilizers can also cause negative effects, such as soil acidification, change of soil microbial diversity, soil compaction, and groundwater pollution (Vance, 2001). Biological nitrogen fixation (BNF) could sustainably supply large amounts of nitrogen for agricultural production and could reduce the application of synthetic nitrogen fertilizer (Ladha and Peoples, 2012). Legumes can recognize and accept various strains of *Rhizobium* to establish a symbiotic relationship; numerous different strains are present in different ecoregions (Zimmer et al., 2016). The recognition and acceptance of rhizobia by legumes are a complex process. Secretion of Nod factor from rhizobia induces the curling of root hair tips, rhizobial cells are wrapped by the curling tips, and then start to infect host cells. Rhizobia induce the development of infection threads in host cells by means of which rhizobia can be transported into the root cortical cells (Riely et al., 2013). When rhizobia symbiotically colonize soybean roots, the plants can fix atmospheric nitrogen (Riches et al., 2013). *Sinorhizobium fredii* strain HH103 can nodulate soybean efficiently, is a fast-growing rhizobia similar as model strain *S. fredii* NGR234. In recent years, the genome of *S. fredii* HH103 has been uncovered, and extensive analyses of its genome and transcriptome have paved a good foundation for gene functional characterization (Margaret et al., 2011; Weidner et al., 2012; Vinardell et al., 2015; López-Baena et al., 2016; Pérez-Montaño et al., 2016). Thus, it is an ideal strain for studying the molecular mechanisms symbiosis between soybean and rhizobium.

The establishment of an effective symbiotic interaction is a complex process that requires multiple signal exchanges between the legume and rhizobia (Miwa and Okazaki, 2017). Among those signals, type III effectors (T3E) play vital roles during the infection of the host with rhizobia. T3Es are secreted through the type 3 secretion system (T3SS) and are translocated into host cells. Within the host cells, the effectors change host signaling, including suppressing plant immunity systems and supplying a more favorable environment for rhizobial infection and multiplication. Similar to plant pathogen effectors, some rhizobial T3Es can also induce strong defense responses that suppress rhizobial infection after being recognized by host legume resistance proteins (Marie et al., 2003; Tampakaki, 2014; Staehelin and Krishnan, 2015; López-Baena et al., 2016). These findings support that T3Es from rhizobia could have either positive or negative influences on the establishment of symbioses. To date, 12 nodulation outer proteins (Nops), namely, NopA, NopAA (GunA), NopB, NopC, NopD, NopI, NopJ, NopL, NopM, NopP, NopT, and NopX, have been identified in *S. fredii* strain HH103 (López-Baena et al., 2016; Jiménez-Guerrero et al., 2019). Among these effectors, NopA and NopB are important components of the needle of the T3SS (Saad et al., 2005; Kim and Krishnan, 2014). NopAA (GunA) is a cellulase that is able to break down the soybean cell wall and so promote infection (Jiménez-Guerrero et al., 2019). NopAA increased *GmPR1* expression at an early stage of symbiosis (Jiménez-Guerrero et al., 2019). NopC is secreted into soybean root cells, exerting a positive function during infection (Jiménez-Guerrero et al., 2015a). NopM, a NEL-domain E3 ubiquitin ligase, appears to induce target sumoylation and may dampen the flg22-induced burst in reactive oxygen species in tobacco (Xin et al., 2012). An HH103 T3SS mutant that failed to secrete T3Es altered the expression of *GmPR1*, suggesting that the T3E might be related to the defense response (Jiménez-Guerrero et al., 2015b). NopP is a substrate for plant kinases, and its secretion by strain USDA112 was associated with host effector-triggered immunity to regulate symbiotic incompatibility with *Rj2* soybeans (Sugawara et al., 2018). Different effectors might have various functions during the establishment of symbiosis.

Rhizobial TtsI can regulate Nops expression during rhizobial infection. In a TtsI mutant, Nops expression was clearly suppressed (López Baena et al., 2008). NopD was first detected in culture supernatants of *S. fredii* strain HH103 induced by genistein (Rodrigues et al., 2007) and was regulated by TtsI (López-Baena et al., 2016). NopD showed homology to Blr1693, a putative outer protein of *Bradyrhizobium japonicum*. The C-terminal region of Blr1693 contains a domain with homology to the ubiquitin-like protease Ulp1 (Kaneko et al., 2002). XopD, one of the *Xanthomonas campestris pv. vesicatoria* T3E, belongs to the C48 cysteine peptidase family and encodes a ubiquitin-like protease 1 (Ulp1). Interestingly, bioinformatic analysis showed that the C-terminal region of *S. fredii* HH103 NopD shares sequence similarities with Blr1693 and XopD. XopD interacts with a small ubiquitin-like modifier (SUMO)-conjugated protein and removes the SUMO conjugate in plants during *X. campestris pv. vesicatoria* infection (Hotson et al., 2003), suggesting that NopD protease might similarly cleave SUMO modifications from SUMO-conjugated proteins. Besides desumoylation, XopD can play a role in host plant defense by interacting with the transcription factor MYB30 (Canonne et al., 2011). In tomato, XopD targeted the transcription factor SlERF4. This interaction influenced signaling in response to ethylene and promoted pathogen reproduction (Kim et al., 2013). *S. fredii* HH103 NopD is one of the Ulp1 proteins with similarities to XopD from *Xanthomonas*. NopD might influence the host cell signaling pathway in a similar way to XopD. However, no proteins that directly interact with NopD have yet been reported.

Numerous important traits in crops have been studied using quantitative trait locus (QTL) mapping to identify genes related to the target traits. Traits related to nodulation are controlled by various genes, and QTL mapping has been used to identify loci or genes associated with symbiosis (Hwang et al., 2014). Several loci related to nodulation have been mapped, such as *rj1, rj2, rj3, rj4, rj5, rj6, rj7*, and *rj8* (Caldwell, 1966; Vest, 1970; Vest and Caldwell, 1972; Caetano-Anollés and Gresshoff, 1991; Vuong et al., 1996). Among these loci, *rj2* and *rj4* were recently cloned (Yang et al., 2010; Tang et al., 2016). The *rj2* gene can associate with NopP to determine symbiotic specificity (Okazaki et al., 2013; Sugawara et al., 2018). *Rj4* can regulate soybean compatibility and incompatibility with rhizobia, and interestingly, the *Rj4* gene was found to associate with some T3Es of *Bradyrhizobium elkanii* to influence establishment of the symbiosis (Faruque et al., 2015). In recent studies in soybean, *PP2C-related gene* and *RPK* were detected by QTL mapping and were shown to interact with *S. fredii* HH103 NopL to regulate the infection of soybean root cells by rhizobia (Zhang et al., 2018). *Via* QTL mapping, *S. fredii* HH103 NopP was found to induce the expression of *TLP* and *MAPK3* during rhizobium infection (Wang et al., 2018). The identification and study of T3Es and their interacting genes could enhance the understanding of the signal communication between host plants and rhizobia during the establishment of symbiosis.

In this study, we show that NopD can be secreted from *S. fredii* HH103 in the presence of genistein. The nodulation effect of NopD was analyzed on 10 soybean germplasms, including Charleston, Dongnong594, Suinong14, and ZYD00006. These varieties showed significant differences in nodulation phenotype after being inoculated with *S. fredii* HH103 (wild type), the NopD and TtsI mutants, respectively. The recombinant inbred line (RIL) population derived from Charleston × Dongnong594 was used to identify QTL loci related to NopD. The conditional QTL related to NopD were verified by the chromosome segment substitution lines (CSSLs). Finally, two genes *Glyma.19g068600* (FBD/LRR) and *Glyma.19g069200* (PP2C) located on the overlap region of QTL location were identified as candidate genes that can interact with NopD. The expression of both genes can be induced by NopD. However, the haplotype effect on nodule traits is different between *19g068600* and *Glyma.19g069200*.

## Materials and Methods

### Strains, Primers, and Plasmids in This Study

Bacterial strains *S. fredii* HH103, the derived mutants HH103Ω*NopD*, HH103Ω*TtsI*, and *Escherichia coli* DH5α were used are listed in Supplementary Table S2. Primers for gene cloning and qRT-PCR are listed in Supplementary Table S1. Plasmids used for mutant construction and studies of gene function are listed in Supplementary Table S2.

### Construction of the HH103Ω*NopD* and HH103Ω*TtsI*

The construction of insertion mutants was performed as follows: a 1.4 kb fragment containing a 550 bp fragment upstream of the *NopD* ATG codon and an 850-bp fragment downstream of the ATG codon was cloned into pGWC, yielding plasmid pGWC-*NopD*1400. A *Spe*I restriction enzyme site was constructed close to the start codon of *NopD* using the Fast Mutagenesis System (Transgene Co., Beijing, China). Primers for site-directed mutagenesis are listed in Supplementary Table S1. A kanamycin Ω interposon was obtained from pEASY-Blunt with *Spe*I and then ligated into pGWC-*NopD*1400 *Spe*I site, yielding pGWC-*NopD*2400Ω. The 2,400 bp construct was then cloned into the suicide vector pJQ200SK (Quandt and Hynes, 1993) using *Xba*I and *Sma*I. The triparental mating was used to the transferred pJQ-*NopD*2400Ω from *E. coli* DH5α cells into *S. fredii* HH103 in the presence of pRK2013 helper plasmid (Figurski et al., 1979). Candidate mutant recombination colonies were obtained by screening for kanamycin resistance and growth on sucrose (5% w/v). Subsequently, positive mutants were screened by antibiotics and 5% sucrose. The candidate NopD and TtsI mutants were detected by PCR, qRT-PCR, and analysis of nodulation outer proteins. All the bacterial strains, primers, and plasmids used for mutant construction are shown in Supplementary Tables S1, S2.

### RNA Isolation of Rhizobia and qRT-PCR Analyses of NopD

*S. fredii* strains HH103, HH103Ω*NopD*, and HH103Ω*TtsI* were incubated with shaking at 28°C in YM medium in the presence or absence of 3.7 μM genistein. Rhizobial RNA was extracted as described (Jiménez-Guerrero et al., 2015a), gDNA was treated by gDNA remover (Transgene Co., Beijing, China) to eliminate its effects on expression, and then RNA samples were synthesized into cDNA using TransScript^®^ One-Step cDNA Synthesis SuperMix (Transgene Co., Beijing, China). qRT-PCR was performed with TransStart^®^ Top Green qPCR SuperMix (Transgene Co., Beijing, China) in a Roche LightCycler 480 II System. The qRT-PCR program was as follows: denaturation at 94°C for 30 s, followed by 40 cycles of 94°C for 5 s, 60°C for 15 s, and 72°C for 10 s. The 16S rRNA gene was used as a reference gene to calibrate the transcript abundance values among different cDNA samples (Crespo-Rivas et al., 2007). The threshold cycle values were analyzed by the software in the Roche LightCycler 480 II. All sample harvests were performed with three biological replicates, and the individual values for each RNA sample were analyzed by three technical replications. The primers for expression analysis are listed in Supplementary Table S1.

### Analysis of NopD in Nodulation Outer Proteins

The wild-type strain and two mutants were each cultured in 500 ml YM medium at 28°C until OD_600_ reached 0.6. The bacteria were cultured in the presence of 3.7 mM genistein for about 40 h at 28°C. After centrifugation of the cells at 8,000 g for 30 min (4°C), the supernatant was collected for purification of outer proteins. To eliminate contamination by bacteria and increase the protein concentration, the supernatant was filtered through Millipore^TM^ filter units (0.22 μM) (Millipore Co., Germany), then concentrated using Millipore^TM^ Amicon^TM^ Ultra-15 (100 kDa) centrifugal filter units (Millipore Co., Germany). Proteins were precipitated in the presence of 10% w/v trichloroacetic acid for 20 h at 4°C then collected by centrifugation at 10,000 g for 20 min (4°C). After washing twice with cold 80% acetone (v/v), the precipitated proteins were resuspended in 8 M urea. Extracellular proteins from the different strains were separated by sodium dodecyl sulfate–polyacrylamide gel electrophoresis (SDS-PAGE). For immunostaining, extracellular proteins were transferred onto nitrocellulose membranes, then the membranes were blocked with TBST pH 7.5 (per liter contains 3.03 g Tris, 8 g NaCl, and 1 ml Tween 20) containing 5% (w/v) skim milk, followed by incubation for 1 h with anti-NopD rabbit antiserum (1,000-fold dilution). Subsequently, the membranes were incubated with goat anti-rabbit immunoglobulin AP-conjugated secondary antibody (Abmart Co., China) for 1 h in accord with the supplier’s instructions, and reaction results were visualized using SuperSignal West Pico Chemiluminescent Substrate (Thermo Co., United States).

### *Agrobacterium tumefaciens*-Mediated Transformation

*Agrobacterium*-mediated transformation by agroinfiltration was performed as follows: the *NopD* gene (GenBank: CEO91485.1) was cloned into the entry vector pGWC, and the entry clone was subsequently recombined into the destination vector pGWB17 using the Gateway^®^ system (Invitrogen Co., United States). Plasmids pGWB17-*NopD*, the empty vector pGWB17-T, and pGWB17-*HopQ1* were transformed into *A. tumefaciens* EHA105 by electroporation. Four-week-old *Nicotiana benthamiana* plants were used for transient expression: the *A. tumefaciens* culture was adjusted to OD_600_ 0.5 using infiltration buffer (10 mM MgCl_2_, 10 mM MES-KOH pH 5.6, 150 μM acetosyringone). Top leaves were used for infiltrating, then at 0–4 days after infiltration, leaves were harvested for detection of cell death. Staining of *N. benthamiana* leaves was performed with trypan blue as described by Tennant (1964). Electrolyte leakage was used to evaluate cell death in leaf tissues by measuring ion conductivity (Mackey et al., 2002).

### Nodulation Tests

For nodulation tests, the wild-type strain *S. fredii* HH103 and mutants HH103Ω*NopD* and HH103Ω*TtsI* were used. The soybean germplasms of different ecoregions and RIL population used are listed in Supplementary Table S3. Soybean seeds were sterilized with chlorine gas for 12–14 h, and then germinated into sterilized 300 ml plastic jar units containing nitrogen-free nutrient solution in the lower vessel. Each jar was kept in a greenhouse with 16 h/8 h light/dark at 26°C until plants grew to Vc stage, then plants were inoculated with 2 ml of 10 mM MgSO_4_ containing approximately 1 × 10^9^ bacteria (Zhang et al., 2018). Thirty days after inoculation, all soybean roots were harvested for nodulation evaluation. The nodulation tests on each soybean germplasm were performed with five replicates and three independent experiments. The statistical significance of differences in phenotype was detected using *t*-tests.

### The Conditional Quantitative Trait Locus Mapping of Nodulation-Related Traits

The experimental RIL population used in this study (*n* = 150) was derived from the cross of Charleston and Dongnong594 (Jiang et al., 2018). A high-density genetic map with 5,308 specific locus amplified fragment sequencing (SLAF-seq) markers had been constructed *via* this population (Qi et al., 2014). Recently, we also completed the genomic resequencing of CSSLs and their parents (unpublished). These genetic backgrounds supply a useful support to identify the candidate genes in interested QTL.

To detect the QTL underlying nodule-related traits, a composite interval mapping method was used with WinQTL Cartographer (Wang et al., 2018; Zhang et al., 2018). The detailed parameters followed the published method (Zhang et al., 2018). The nodule number (NN) and nodule dry weight (NDW) of RILs and their parents after inoculation with NopD mutant or the parental strain were used for QTL identification. The differences in phenotypic values were used to determine the location of conditional QTL. At the same time, the phenotype differences in RILs inoculated with HH103Ω*TtsI* or the parental strains were used to determine the conditional QTL’s location (Wang et al., 2018). The detailed method is as follows. When the operation was running, the control marker number was set to 5 and window size was 10 cM. The forward regression method and a walk speed of 0.5 cM were selected. The proportional and additive effects of variances interpreted by each specific QTL were obtained by composite interval mapping analysis. The log of the odds (LOD) score peaks higher than 3.0 (WinQTL Cartographer default threshold) was selected to indicate the existence of conditional QTL for the nodule traits inoculated with the two type strains, respectively. About the additive-effects signals, “+” indicates increasing allelic effects from “Dongnong 594” and “−” indicates decreasing allelic effects from “Charleston.” The 1,000 permutations of each genotypic marker against the phenotype in RIL population determined the experimental threshold levels for linkage. When the two values for a marker were greater than the critical value at *p* = 0.05, it indicated that the linkage was significant. CSSLs produced by the cross between soybean cultivar SN14 and wild soybean ZYD00006 (*G. soja* Sieb. & Zucc.) were used for verifying consensus QTL, according to the genetic map by Xin et al. (2016).

### Annotation of Candidate Genes in Quantitative Trait Locus of NopD-Related

The “Williams 82. a2. v1” genome was the first published soybean genome and could provide valuable information for QTL mapping of soybean important traits (Brensha et al., 2012). Phytozome website^1^ and Soybase database^2^ integrated the soybean Williams 82 genome information and the latest information on the soybean genome uploaded by researchers, could provide necessary information for mapping. Our laboratory completed the construction of a high-density genetic map of the RIL population in the early stage (Qi et al., 2014). So the soybean genes in the major QTL intervals could be identified by combining the high-density genetic map information with two important database tools, and corresponding gene annotations were performed (Wang et al., 2018). Candidate genes involved in the plant immunity and signal conduction were selected from the annotation data, which were used for subsequent verification.

### Verification of NopD Candidate Genes by qRT-PCR

qRT-PCR in various soybean germplasm materials was performed to verify candidate genes that potentially interacted with NopD. Root samples were harvested at 0, 12, 24, 36, 48, and 60 h post-inoculation with the wild-type strain HH103, NopD mutant, and TtsI mutant, respectively. The classic TRIzol reagent (Invitrogen Co., United States) was used for the extraction of total RNA from soybean roots, and total RNA was treated by gDNA remover (Transgene Co., Beijing, China) to eliminate its effects on expression, and then RNA samples were synthesized into cDNA by the TransScript^®^ One-Step cDNA Synthesis SuperMix (Transgene Co., Beijing, China). qRT-PCR was performed as described above, and *GmELF1b* was used as the internal control to normalize the transcript amounts in different samples (Jian et al., 2008). The gene-specific primers for qRT-PCR are listed in Supplementary Table S1.

### Haplotype Analysis of Candidate Genes Based on Chromosome Segment Substitution Line Population

Haplotype analysis of *Glyma.19g068800* and *Glyma.19g069200* was based on the resequencing data of CSSL populations in a subset of 142 soybean accessions. The genomic regions including the gene transcript sequence and approximately 2.0-kb promoter region of two genes upstream of the start codon were used for the haplotype analysis.

## Results

### NopD Can Be Secreted From *S. fredii* HH103 by Type 3 Secretion System

**FIGURE 1:**
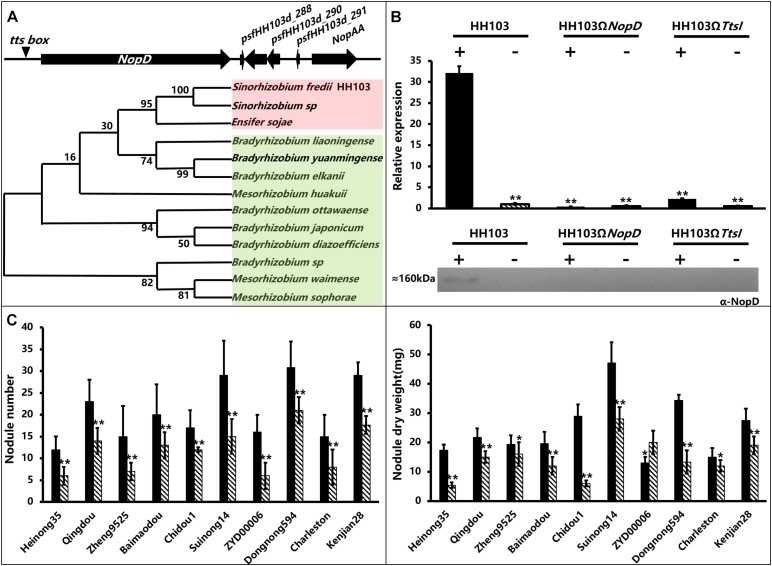
The information of NopD gene, detection of NopD protein induction by genistein and nodule traits of soybean plants inoculated with *S. fredii* wild-type HH103 or mutants HH103Ω*NopD* and HH103Ω*TtsI.* **(A)** Position of the NopD gene and phylogenetic tree of NopD from various rhizobial strains by neighbor-joining method using MEGA6.0 software. **(B)** Expression analysis of NopD in *S. fredii* wild-type strain HH103 and mutants HH103Ω*NopD* and HH103Ω*TtsI* with (+) or without (−) genistein (3.7 μM). Final expression was calculated relative to the expression of the wild-type strain HH103 in the absence of genistein. All sample harvests were performed with three biological replicates, and the individual values for each RNA sample were analyzed by three technical replications. The sample of the wild-type strain *S. fredii* HH103 in the absence of genistein was used as the control. Asterisks indicate significant differences at the level α = 1% (*P* < 0.01). Immunoblot analysis of NopD in extracellular protein extracts of the wild-type strain *S. fredii* HH103 and indicated mutant derivatives induced with or without genistein (3.7 μM). Immunoblots were performed with anti-NopD antibodies. **(C)** The analysis of phenotype was performed three times; significant differences were determined by *t*-tests; * indicates 0.01 ≤ *P* ≤ 0.05 and **indicates *P* ≤ 0.01. Soybean varieties (with origins): Heinong35 (Heilongjiang), Qingdou (Shanxi), Zheng9525 (Henan), Baimaodou (Zhejiang), Chidou1 (Inner Mongolia), Suinong14 (Heilongjiang), ZYD00006 (Heilongjiang), Charleston (America), Dongnong594 (Heilongjiang), Kenjian28 (Heilongjiang).

The *NopD* coding sequence was located in the plasmid pSfHH103d of *S. fredii* HH103. The promoter region contains a *tts* box (−257 bp), supporting the idea that *NopD* can be induced by TtsI (Figure 1A). By phylogenetic analysis, we can separate the NopD tree into two branches (I-Red and II-Green). Branch I comprises only three *Sinorhizobium* species. Branch II comprises *Mesorhizobium* and *Bradyrhizobium* species without *Sinorhizobium* species (Figure 1A).

*NopD*’s expression was studied by qRT-PCR in strains HH103, HH103Ω*NopD*, and HH103Ω*TtsI* induced or not by genistein. The qRT-PCR results showed that genistein promoted the expression of *NopD* significantly in the wild strain (Figure 1B), but *NopD* expression was not detected in the NopD and TtsI mutant either with or without genistein. This result was consistent with the previous reports that *NopD*’s expression was downregulated in a flavonoid mutant, NodD1 mutant, and TtsI mutant (Pérez-Montaño et al., 2016). An antibody against NopD was used to detect the NopD protein in supernatants from HH103, HH103Ω*NopD*, and HH103Ω*TtsI*. Western blot results showed a band corresponding to NopD (about 160 kDa) in samples from *S. fredii* HH103 induced with genistein, but not in NopD or TtsI mutants (Figure 1B).

### Nodulation Tests

To elucidate the role of NopD in establishing symbiosis, we collected 10 soybean varieties with differing genetic backgrounds from various ecoregions in China and United States. In most soybean germplasms, there were significant differences in NN and NDW after inoculation with the NopD mutant or the wild-type HH103 (Figure 1C). Only in Kenjian28, NopD had a negative effect on the NN and NDW, and there was no difference in NN of Charleston when inoculated with the wild strain or HH103Ω*NopD*. However, NopD had a positive effect on NN and NDW in eight of the 10 soybean germplasms, except for the NDW of ZYD00006 (Figure 1C). Thus, NopD exerting either positive or negative effects on the various soybean germplasms might be due to the different genetic backgrounds of germplasms. On the other hand, these results support NopD mainly played a positive effect on soybean nodulation.

Nodulation tests show that Dongnnong594 and Charleston, as the parents of RIL population, had a significant difference in NN and NDW when inoculated with wild strain and NopD mutant, the same situation also occurs in the CSSL populations with Suinong14 and ZYD00006 as their parents, so RIL and CSSL populations could be used to map important QTL interactions with NopD.

### NopD Induces Leaf Death in *Nicotiana benthamiana*

**FIGURE 2:**
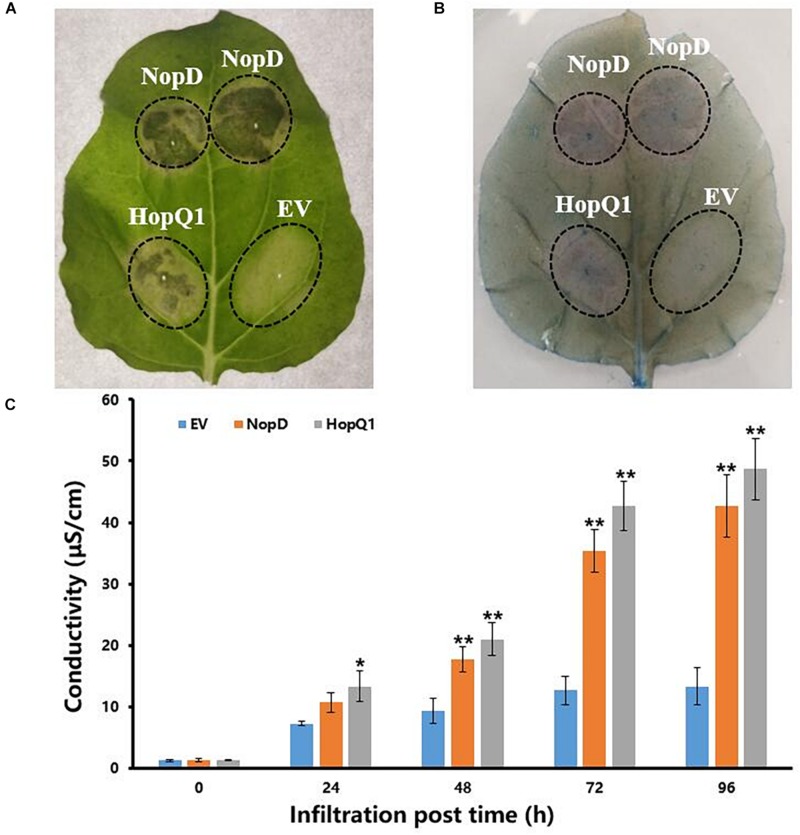
Effects of NopD on *N. benthamina* cells. Infiltrated zones are marked by circles (EV, infiltration zone with *A. tumefaciens* carrying the empty vector pGWB17-T). For comparison, *A. tumefaciens* carrying plasmid pGWB17-HopQ1-myc was also used for infiltration (expression of HopQ1 of *P. syringae* pv. tomato DC3000). **(A)** Leaves were photographed 4 days post infiltration. **(B)** Trypan blue-based cell death staining of a representative *N. benthamiana* leaf transiently expressing NopD and HopQ1 4 days post *Agrobacterium infiltration*. **(C)** Electrolyte leakage levels in leaves. “*” indicated the significant differences (*p* ≤ 0.05) and “**” indicated the significant differences (*p* ≤ 0.01).

*Agrobacterium*-mediated transient transformation was used to study NopD’s effects within tobacco leaves. pGWB17, a binary vector containing the cauliflower mosaic virus 35S promoter and the NopD coding sequence, was introduced into *A. tumefaciens* EHA105, which was then infiltrated into leaves of 4-week-old tobacco plants. Four days after infiltration, a clearly necrotic leaf zone (hypersensitive response with induced cell death) was observed in tissue transformed with NopD as well as in leaves expressing HopQ1 of *Pseudomonas syringae*, which was used as a positive control (Figure 2). These results indicated that NopD functions like an avirulence effector in tobacco. Trypan blue staining and electrolyte leakage were used to detect the effect of NopD expression in leaves (Figure 2).

### Phenotype of Nodulation Analysis in Recombinant Inbred Lines

Nodulation tests on the soybean germplasms showed that there were significant differences in NN and NDW between Charleston and Dongnong594 when inoculated with the wild-type strain HH103 or NopD and TtsI mutants (Figure 1C and Table 1). The RIL population was derived from the cross between Charleston and Dongnong594. From the nodulation tests, the NopD mutant gave a higher average NN and NDW in the whole RIL population compared with inoculation with the wild-type strain or TtsI mutant. There were no significant differences in average NN or NDW of the whole RIL population after inoculation with the wild-type strain or the TtsI mutant. The RIL population has a more complex genetic background than the individual parents Charleston and Dongnong594, the genetic information between individuals were quite different, these individuals should have different responses to the wild strain, NopD mutant, or TtsI mutant, and this might have caused the observed differences compared with 10 soybean varieties in phenotype. So the nodule traits of the whole RIL population are not representative after inoculation with the wild strain, NopD mutant, and TtsI mutant. The more complex genetic background of the RIL population should facilitate the mining of QTL associated with the phenotype.

**TABLE 1:** Nodule traits of the recombinant inbred line (RIL) population inoculated with the wild-type strain and NopD and TtsI mutants.

	**RILs (*n* = 150)**			**Parents (average)**	
	**Traits**	**Average**	**Standard deviation**	**Charleston**	**Dongnong594**
HH103 Rif^R^	Nodule number	8.1	10.9	20.6 ± 4.7**	30.8 ± 5.8
	Nodule dry weight (mg)	7.5	14.9	18.0 ± 3.6**	24.3 ± 3.3
HH103 Rif^R^Ω*NopD*	Nodule number	11.2*	7.0	13.4 ± 3.5**	17.8 ± 4.1
	Nodule dry weight (mg)	9.9*	18.4	12.0 ± 2.9**	21.3 ± 4.6
HH103 Rif^R^Ω*TtsI*	Nodule number	7.0	4.4	12.2 ± 3.8**	18.4 ± 4.7
	Nodule dry weight (mg)	7.3	7.2	13.0 ± 3.6**	19.3 ± 5.8

*Indicates significant differences with different inoculations, p ≤ 0.05, **indicates p ≤ 0.01.

### Conditional Quantitative Trait Locus Mapping for Nodule Number and Nodule Dry Weight and Validation of Consensus Quantitative Trait Locus

**FIGURE 3:**
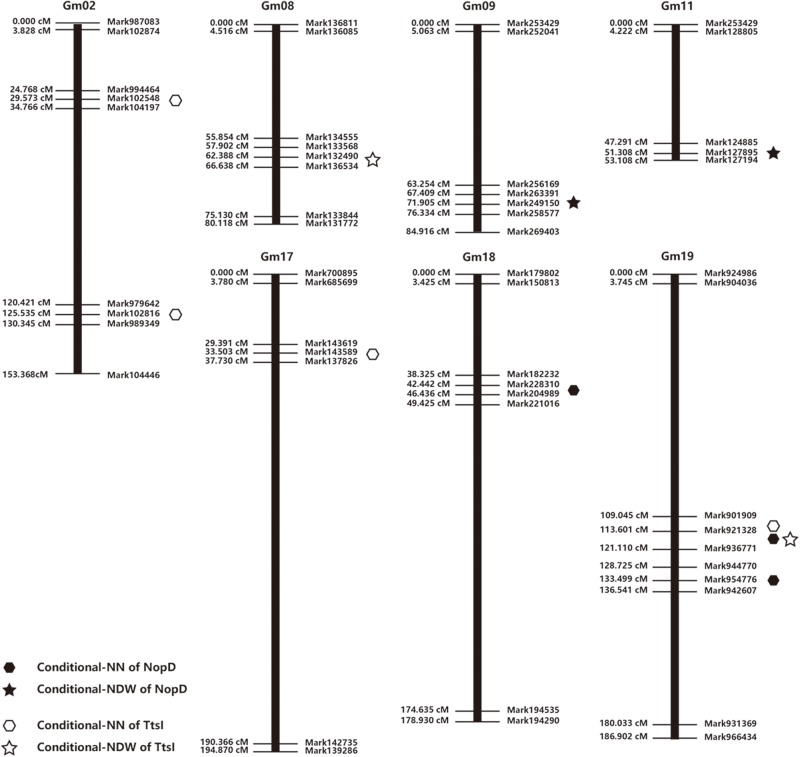
Distribution of conditional quantitative trait locus (QTL) for nodule number and nodule dry weight among linkage groups and chromosomes.

Seven conditional QTL for NN and four conditional QTL for NDW were identified, after inoculation with *S. fredii* HH103 or the NopD and TtsI mutants (Table 2 and Figure 3). Seven conditional QTL associated with NN were located on chromosomes Gm02 (*n* = 2), Gm17 (*n* = 1), Gm18 (*n* = 1), and Gm19 (*n* = 3), and four conditional QTL for NDW were located on Gm08 (*n* = 1), Gm9 (*n* = 1), Gm11 (*n* = 1), and Gm19 (*n* = 1).

**TABLE 2:** Distribution of conditional quantitative trait locus (QTL) for nodule number (NN) and nodule dry weight (NDW) among linkage groups and chromosomes.

**Strain**	**Trait**	**LG/QTL**	**Chrom.**	**Start position (cM)**	**End position (cM)**	**LOD^a^**	***R*^2^ (%)^b^**	**ADD^c^**
HH103Ω*NopD*	NDW	QK/NDW01	9	68.81	71.81	3.00	5.78	0.49 × 10^–3^
		QB1/NDW02	11	50.31	53.31	3.70	9.36	0.64 × 10^–3^
	NN	QG/NN01	18	42.91	45.91	3.30	2.08	−1.21
		QL/NN02	19	131.73	134.73	3.80	0.62	−1.32
		QL/NN03	19	116.92	119.92	3.20	1.83	−1.10
HH103Ω*TtsI*	NDW	QA2/NDW03	8	61.41	64.41	3.50	1.31	0.95 × 10^–3^
		QL/NDW04	19	116.92	119.92	3.90	5.38	1.78 × 10^–3^
	NN	QD1b/NN04	2	28.11	31.11	3.90	6.47	−1.71
		QD1b/NN05	2	124.32	127.32	4.80	3.70	1.73
		QD2/NN06	17	31.81	34.8	3.60	7.12	−1.59
		QL/NN07	19	109.52	112.52	4.00	2.53	0.95

**^*a*^LOD, log of odds. ^*b*^R^2^ (%), the contribution rate of the QTL. ^*c*^ADD, the additive effects contributed by the QTL. LG, linkage group; QD1b, chromosome 2; QA2, chromosome 8; QK, chromosome 9; QB1, chromosome 11; QD2, chromosome 17; QG, chromosome 18; QL, chromosome 19.**

Overlapping conditional QTL (118.4 cM) for NN and NDW were identified on Chromosome 19, and we found two further conditional QTL (111.0 and 133.2 cM) close to these overlapping QTL. An earlier investigation of soybean revealed two QTL (99.7 and 108 cM) on Gm19 associated with nodule weight, and two QTL (97.5 and 108.2 cM) associated with nodule size (Hwang et al., 2014). This previously identified QTL support that in the region of QTL we identified is confident. So we selected this region as a candidate region that might contain gene response to NopD. To test our hypothesis, specific lines of CSSLs were identified and used for further work.

**FIGURE 4:**
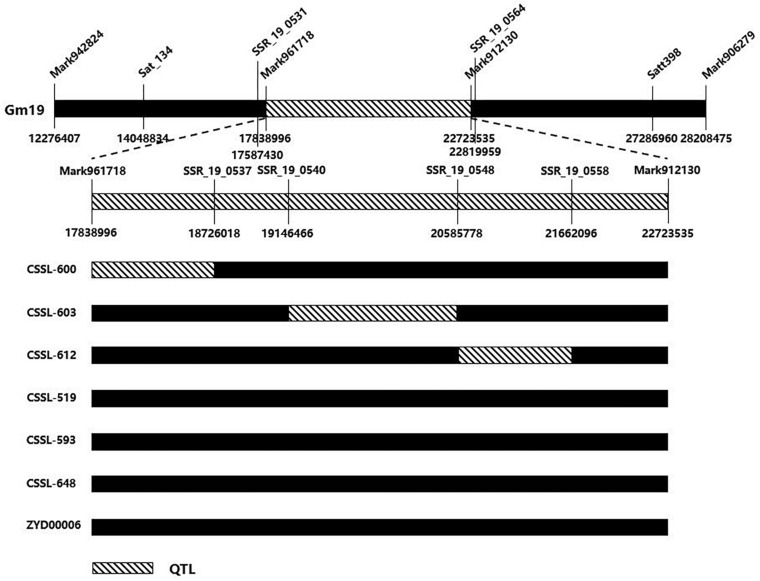
Validation of consensus quantitative trait locus (QTL) in chromosome segment substitution lines (CSSLs). The consensus QTL associated with nodulation phenotype on chromosome Gm19 (97.5–133.2 cM) had a corresponding partial region in the substituted wild soybean chromosomal segment on Gm19 in CSSL-600, CSSL-603, and CSSL-612. On the contrary, there was no corresponding region in CSSL-519, CSSL-593, CSSL-648, or ZYD00006.

Nodulation tests showed significant differences in NN and NDW between Suinong14 and ZYD00006 after inoculation with wild-type strain HH103, NopD mutant, and TtsI mutant (Figure 1C). A CSSL population derived from the cross between Suinong14 and ZYD00006 was used to verify whether the overlapping QTL might be related to NN or NDW in other soybean populations (Supplementary Table S4). We selected six soybean lines from the CSSL, three of which (CSSL-600, CSSL-603, and CSSL-612) had higher NN and NDW than the parent Suinong14 after inoculation with NopD mutant and TtsI mutant, and another three of which (CSSL-519, CSSL-593, and CSSL-648) had lower NN and NDW than ZYD00006 after inoculation with NopD mutant and TtsI mutant. SSR markers located the conditional QTL on chromosome 19 when the RIL population were screened. Substituted chromosome fragments were also detected in related regions: we found an overlap region between the conditional QTL and the fragment of a CSSL (Sat_134–Satt398) on chromosome 19 (97.5–133.2 cM), these lines including CSSL-600, CSSL-603, and CSSL-612. No corresponding region was found in lines CSSL-519, CSSL-593, CSSL-648, or the parent ZYD00006 (Figure 4).

Furthermore, our candidate QTL were close to those QTL in the previous reports and identified by the CSSL population. No QTL or genes interacting with NopD had been identified in previous reports, so it was interesting to determine whether the QTL identified in our work might interact with NopD.

### Expression Analysis of Candidate Genes Associated With Nodulation Phenotype

**FIGURE 5:**
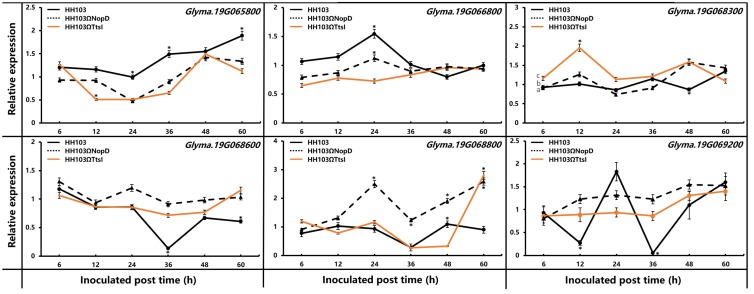
Relative expression of *Glyma.19g065800, Glyma.19g066800, Glyma.19g068300, Glyma.19g068600, Glyma.19g068800*, and *Glyma.19g069200* was measured by qRT-PCR in RNA extracted from roots of soybean Suinong14 plants inoculated with *S. fredii* HH103 wild-type or mutants HH103Ω*NopD* and HH103Ω*TtsI*. Uninoculated Suinong14 plants were used as the control. The 2^–ΔΔ*Ct*^ method was used to calculate the relative expression levels of candidate genes. Error bars indicate the mean ± standard error of three replications. Significant differences were determined by *t*-tests, and “*” indicated the significant differences (*p* ≤ 0.05) at the time point.

In the confident QTL region, seven genes (*Glyma.19g065800, Glyma.19g066800, Glyma.19g067200, Glyma.19g068300, Glyma.19g068600, Glyma.19g068800*, and *Glyma.19g069200*) on Chromosome 19 associated with pathogen resistance, signal exchange, and symbiosis were selected for further analyses (Supplementary Table S5). To identify whether these genes have interaction with NopD, soybean Suinong14 was inoculated with the wild-type HH103, HH103Ω*NopD*, and HH103Ω*TtsI*. The expression pattern of these genes was detected by qRT-PCR (Figure 5). Among the seven genes, *Glyma.19g067200* showed no expression in Suinong14 inoculated with any of the three strains because of no expression signal detected by qRT-PCR. At 12 h post-inoculation, the *Glyma.19g065800* expression levels were significantly different comparing TtsI mutant with the wild strain and NopD mutant, at 24 and 60 h post-inoculation, the wild strain could induce the higher expression. It showed that NopD and TtsI mutant could induce a different expression pattern, indicating that *Glyma.19g065800* had no obvious interaction with NopD. At 24 h post-inoculation with the wild-type strain HH103 and NopD mutant, the expression level of *Glyma.19g066800* reached a maximum, but TtsI mutant did not have a similar trend. *Glyma.19g068300* had the similar expression trend in soybean inoculated with three strains, and expression levels all changed around 1.0. At 12 h post-inoculation, the *Glyma.19g068300* expression levels was significantly different inoculated with TtsI mutant compared to the wild strain and NopD mutant. These results showed that *Glyma.19g068300* and *Glyma.19g066800* had no obvious interaction with NopD similar with *Glyma.19g065800*. The expression of *Glyma.19g068800* had a strange pattern; NopD mutant could induce the gene to reach two peaks at 24 and 60 h post-inoculation, its expression level was higher compared with inoculation with the wild strain and TtsI mutant which had a similar induction pattern, so we could not infer that *Glyma.19g068800* could interact with NopD. *Glyma.19g068600* and *Glyma.19g069200* showed significantly different expression patterns in Suinong14 after inoculation with *S. fredii* HH103 and the two mutant strains. When Suinong14 was inoculated with *S. fredii* HH103, the expression patterns of *Glyma.19g068600* and *Glyma.19g069200* were similar. At 36 h post-inoculation with the wild-type strain HH103, the expression level of *Glyma.19g068600* and *Glyma.19g069200* reached a minimum, and then the expression level of these two genes increased. But in a clear difference from the wild-type strain, infection with the mutants HH103Ω*NopD* and HH103Ω*TtsI* showed no evident effects on the expression patterns of these two genes. At 36 h post-inoculation with HH103Ω*NopD* and HH103Ω*TtsI*, the expression level of *Glyma.19g068600* was 6.7 and 5.2 times higher than expression induced by inoculation with HH103. Under the same conditions, the expression level of *Glyma.19g069200* induced by HH103Ω*NopD* and HH103Ω*TtsI* was 10.7 and 12.0 times higher than that in plants induced by HH103, respectively. These qRT-PCR results supported the hypothesis that NopD interacted with *Glyma.19g068600* and *Glyma.19g069200* in the early stage of rhizobial infection.

### Haplotypes of *Glyma.19g068600* and *Glyma.19g069200* That Correlated With Nodulation Traits

**FIGURE 6:**
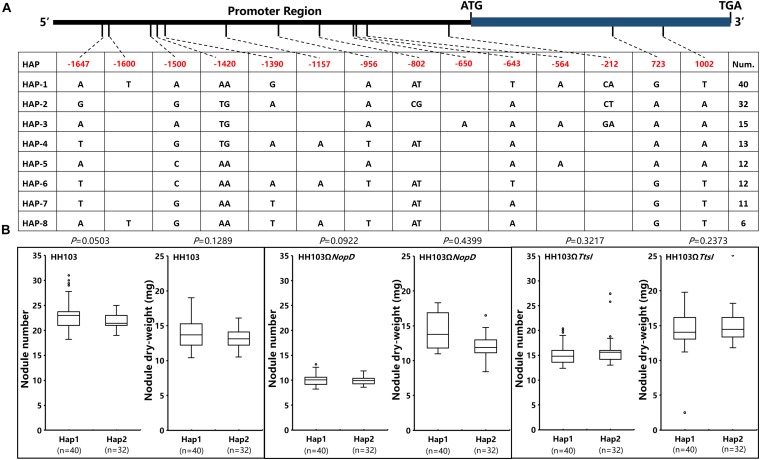
The analysis of *Gm19g068600* haplotypes and nodule traits in two Hap accessions. **(A)** *Gm19g068600* gene haplotypes (eight haplotypes) in chromosome segment substitution line (CSSL) populations (*n* = 142). The start codon site is set at “0” position. **(B)** Comparison and analysis of the nodule phenotype between haplotypes Hap1 (*n* = 40) and Hap2 (*n* = 32). n represents the number of the two haplotypes. Two-tailed *t*-test was used to detect the significance.

Considering that *Gm19g068600* and *Gm19g069200* can be suppressed at a lower level in Suinong14 during the wild HH103 infecting (Figure 5) and nodule traits had the significant differences in parents of CSSL populations (Figure 1C). We analyzed the haplotypes of *Gm19g068600* and *Gm19g069200* in 142 accessions of CSSL populations. In total, 14 single-nucleotide polymorphisms (SNPs) and/or indels were found in the promoter and coding sequence of *Gm19g068600* from the CSSL populations. According to 14 SNPs and/or indels, 142 soybean accessions were classified into eight haplotypes (Hap1–Hap8) (Figure 6A). Hap1, the largest group, includes 40 soybean accessions; Hap2 including 32 soybean accessions was the second largest group. In these two types, two SNPs are located in the exon, but these differences did not result in the amino acid change. Ten SNPs and indels located in the promoter sequence. The relative expression of gene was detected during some rhizobia strains infecting in Hap1 and Hap2, the relative expression showed that *Gm19g068600* had the similar expression pattern in Hap1 accession and Hap2 accession after inoculated with the wild strain HH103, NopD mutant and TtsI mutant, respectively. The nodule traits of Hap1 and Hap2 accessions were further compared, the nodulation results show that the nodule traits of haplotypes do not have significant differences after inoculation with the wild HH103, NopD mutant, and TtsI mutant, respectively (Figure 6B). The relative expression and nodule trait analysis indicated that *Gm19g068600* could be related to NopD, but haplotype difference could not influence expression pattern.

**FIGURE 7:**
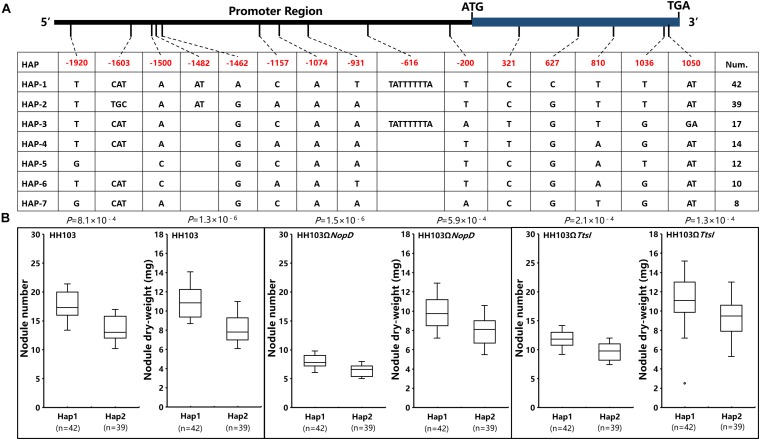
The analysis of *Gm19g069200* haplotypes and nodule traits in two Hap accessions. **(A)** *Gm19g069200* gene haplotypes (seven haplotypes) in chromosome segment substitution line (CSSL) populations (*n* = 142). The start codon site is set at “0” position. **(B)** Comparison and analysis of the nodule phenotype between haplotypes Hap1 (*n* = 42) and Hap2 (*n* = 39). n represents the number of the two haplotypes. Two-tailed *t*-test was used to detect the significance.

**FIGURE 8:**
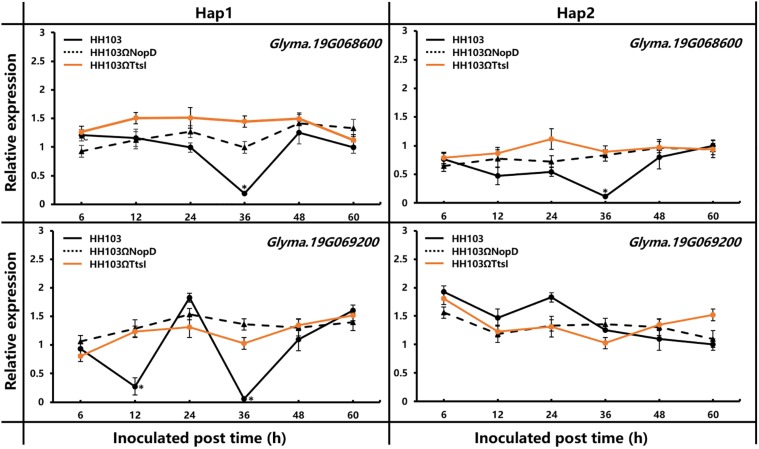
Relative expression of *Glyma.19g066800* and *Glyma.19g069200* was measured by qRT-PCR in RNA extracted from roots of Hap1 and Hap2 accession plants inoculated with *S. fredii* HH103 wild-type or mutants HH103Ω*NopD* and HH103Ω*TtsI*. Uninoculated soybean plants were used as the control. The 2^–ΔΔ*Ct*^ method was used to calculate the relative expression levels of candidate genes. Error bars indicate the mean ± standard error of three replications. Significant differences were determined by *t*-tests, and ‘*’ indicated the significant differences (*p* ≤ 0.05) at the time point.

The haplotype analysis of *Gm19g069200* shows that there were 15 SNPs and/or indels found in the promoter and coding sequence. Based on the 15 SNPs and/or indels, 142 soybean accessions were classified into seven haplotypes (Hap1–Hap7). Hap1, the largest group, includes 42 soybean accessions, and Hap2 was the second largest group, including 39 soybean accessions (Figure 7A). One SNP located in the exon, but the change did not result in the amino acid change, five SNPs or indels located in the promoter sequence. As it is known, the promoters always affects the function of genes by regulating gene expression, and the relative expression of *Gm19g069200* was studied in one Hap1 accession and one Hap2 accession, respectively. These results show that the relative expression of *Gm19g069200* had a significant difference in Hap1 and Hap2 soybean accession (Figure 8). *Gm19g069200* could be regulated by NopD in HH103 *via* Hap1 accession, but not in Hap2 accession. The nodule traits of Hap1 and Hap2 accessions were further compared, and the nodulation results show that haplotypes have significantly different effects on nodule trait after inoculation with the wild strain, NopD mutant, and TtsI mutant. The NN and NDW of Hap1 soybean accessions were higher than the Hap2 soybean accessions, and the difference was significant (*P* < 0.01) (Figure 7B). Similar to the wild strain, NopD mutant and TtsI mutant could result in higher NN and NDW in Hap1 accessions than Hap2 accessions. NopD mutant and TtsI mutant could reduce NN and NDW compared with the wild strain in both Hap1 accessions and Hap2 accessions. These results suggested that *Gm19g069200* can be associated with the rhizobia infection. Comparisons of nodule phenotypes of Hap1 accessions and Hap2 accessions inoculated with different strains show that the presence of NopD in the wild strain could cause a greater difference than the absence of NopD, indicating that NopD could be associated with *Gm19g069200.*

## Discussion

The locus we identified in this study on soybean chromosome Gm08 overlaps with a previously identified QTL related to compatibility of soybean with *Bradyrhizobium* strains (Ramongolalaina et al., 2018). A QTL previously shown to be associated with nodule weight (Hwang et al., 2014) was found to be adjacent to the QTL on Gm18 in our study. In addition, a locus on Gm17 related to NopL, a T3SS effector of *S. fredii* HH103 (Zhang et al., 2018), overlaps with a locus adjacent to a QTL on Gm17 that we detected.

We identified two genes (*Glyma.19g068600* and *Glyma.19g069200*) by qRT-PCR that had a similar expression pattern induced by the wild-type strain HH103 (showing a minimum of expression at 36 h after inoculation), but this pattern was not identified when soybean was inoculated with the mutant HH103Ω*NopD* or HH103Ω*TtsI*. This expression pattern suggests that *Glyma.19g068600* and *Glyma.19g069200* might be associated with the NopD-triggered signaling pathway activated after inoculation with rhizobia. *Glyma.19g068600* encodes a protein belonging to the F-Box/LRR-repeat (FBD/LRR) protein family. FBD/LRR proteins have been identified in several plant species, for instance, in radish (Zhai et al., 2016), soybean (Chen et al., 2018), tomato (Du et al., 2014; Quintana-Camargo et al., 2015), and peanut (Agarwal et al., 2018). Some members of the FBD/LRR protein family have been found to be associated with pathogen resistance, such as tomato FBD/LRR3, where overexpression of *FBD/LRR3* increased plant resistance to *X. perforans* (Du et al., 2014). One novel *FBD/LRR* gene was identified from a high-density genetic map, and expression of this gene was induced during pathogen resistance responses in peanut (Agarwal et al., 2018). Using proteome analysis, GmFBD/LRR was shown to have a different expression pattern in response to *Phytophthora sojae* infection. The FBD/LRR proteins contain an F-box domain and LRR-repeat domain. The F-box domain is usually associated with a transcription factor that participates in the defense response induced by jasmonic acid (Xu et al., 2009). The LRR-repeat domain has been identified in a functionally and evolutionarily diverse series of proteins. In these proteins, the LRR-repeat domains are essential to protein–protein interactions or signal transduction (Tornero et al., 1996). From the nodulation tests in this study, the NopD mutant decreased NN and NDW in most soybean germplasms, suggesting that NopD could play a positive role in rhizobial infection and nodule formation. Our qRT-PCR results confirmed that NopD can depress *FBD/LRR* expression during infection with the wild-type strain HH103. The haplotype analysis classified the haplotypes into eight types, and Hap1 and Hap2 were the major two types, but Hap1 and Hap2 could not change the gene expression pattern and nodule traits inoculated with the same rhizobia strain. The finding that *FBD/LRR* encodes an FBD/LRR protein was unexpected. Since some FBD/LRR proteins in other plants are known to be associated with resistance to pathogens, the interaction of NopD with an FBD/LRR protein is interesting, and so further research will be necessary to clarify the nature of this interaction.

*Glyma.19g069200* encodes a protein phosphatase 2C (PP2C). The PP2C protein family is widely distributed in almost all plants, whether eukaryotic or prokaryotic plant. PP2C proteins interact with many signaling pathways. Overexpression of the rice *PP2C* gene *OsBIPP2C1* in transgenic tobacco was found to suppress pathogen infection and enhance some abiotic tolerance (Hu et al., 2006). GmPP2C3a, a member of the soybean PP2C protein family, was identified as an antiviral protein that was able to suppress virus infection and spread (Seo et al., 2014). Earlier studies indicated that PP2Cs negatively regulate the mitogen-activated protein kinase (MAPK) pathways in yeast and *Arabidopsis thaliana* (Meskiene et al., 2003), as well as the MAPK pathway triggered by a bacterial flagellin (Cristina et al., 2010). The MAPK pathway might be involved in establishing the rhizobial symbiosis since *MtTDY1*, one of the MAPK pathway genes, was shown to be associated with nodule formation and to regulate the development of the root tip (Schoenbeck et al., 1999). In *Lupinus albus*, bradyrhizobia can activate MAPK genes *SIMK* and *SAMK*, and mutants of SIMK and SAMK inhibited bradyrhizobial infection (Fernandez-Pascual et al., 2006). In soybean, *GMK1* has been identified as a MAPK homolog, and its expression was associated with infection by *Bradyrhizobium japonicum* USDA110 (Lee et al., 2008). T3SS is essential to rhizobial infection, and T3Es can also interact with MAPKs during establishment of symbiosis. The T3E NopP can induce MAPK3 expression at an early stage of rhizobial infection (Wang et al., 2018). In specific *Lotus japonicus* lines of a monogenic-recessive mutant carrying the symbiosis-associated locus, considerable changes to *LjNPP2C1* were observed, suggesting that *LjNPP2C1* may be functional during the early and late nodule development stages (Kapranov et al., 1999). NopL, another T3SS effector of HH103, was shown to suppress the expression of the PP2C-related protein *Glyma.07g099700* during HH103 infection of soybean (Zhang et al., 2018). In this study, expression pattern and haplotype analysis of *PP2C* gene were studied; the result showed *PPC2* to be depressed by the wild-type strain HH103 (which produces NopD) in Hap1 accession, but not in Hap2. This also explained that *PP2C* had a different expression regulation pattern because of the difference in promoter region. Nodule traits indicated that there was a significant difference between Hap1 and Hap2 inoculated with the wild strain HH103, NopD mutant, and TtsI mutant. These results suggesting that expression of *PP2C* could regulation nodule formation. It is interesting to detect the interaction mechanism between NopD and PP2C. However, further research is essential to fully clarify this interaction.

The T3Es have been shown to be secreted into legume cells *via* rhizobial T3SSs, in a similar way to many gram-negative pathogenic bacteria. In a previous study, the function of rhizobial T3Es in legume cells has been questioned (Büttner and Bonas, 2006). However, more recent research has confirmed that T3 effectors are essential to symbiosis (Schechter et al., 2010; Wenzel et al., 2010). NopD is a conserved T3Er in most rhizobial strains, and our nodulation tests showed that its secretion can positively influence the formation of nodules. The expression of NopD also induced tobacco leaf cell death, giving us evidence that NopD was directly or indirectly recognized by a non-host plant. In our study, NopD influenced the immunity pathway but had no effect on the symbiosis pathway (Figure 2). This is similar to XopD, one of the *X. campestris pv. vesicatoria* T3SS effectors, which belongs to the C48 cysteine peptidase family. XopD is able to suppress the host immunity and so promote infection by the pathogens. This suggested that NopD could influence the symbiosis between soybean and rhizobia by affecting signal transduction in the host immunity system. We propose that NopD inhibits *FBD/LRR* and *PP2C* expression, thereby promoting infection by the wild-type HH103.

In this work, *FBD/LRR* and *PP2C* were identified by QTL mapping and can be used to aid further detect of signaling communication induced by NopD. Further identification and clarification of the host genes involved in interactions with rhizobial effector molecules could enhance the understanding of symbiosis establishment.

## Data Availability Statement

The raw data supporting the conclusions of this article will be made available by the authors, without undue reservation, to any qualified researcher.

## Author Contributions

DX, QC, and CL conceived the study and designed and managed the experiments. ZQ, HJ, RZ, and XW provided soybean seeds. JinW, JieW, CM, JL, LC, and DY performed trials and collected data. JinW, QK, HZ, JieW, ZS, HLiu, ZZ, JZ, HLi, and QW completed statistical analyses of phenotypic data and wrote the manuscript. DX, QC, and JinW participated in correcting the manuscript. All authors contributed to writing the manuscript.

## Conflict of Interest

The authors declare that the research was conducted in the absence of any commercial or financial relationships that could be construed as a potential conflict of interest.

## Footnotes

1 https://phytozome.jgi.doe.gov/pz/portal.html#

2 https://www.soybase.org/
