# Supplementary material for: QTL Mapping and Data Mining to Identify Genes Associated With the Sinorhizobium fredii HH103 T3SS Effector NopD in Soybean
Source: Front Plant Sci. 2020 May 19;11:453. doi: 10.3389/fpls.2020.00453 (PMC7249737; doi:10.3389/fpls.2020.00453)
Supplement: Supplementary file 1 [file Data_Sheet_1.docx]

**QTL mapping and gene mining to identify genes on soybean associated with the *Sinorhizobium fredii* HH103 T3SS effector protein NopD**

**Jinhui Wang^1,†^****, Jieqi Wang^1,^****^†^****, Chao Ma^1,†^, Ziqi Zhou^1^,·Decheng Yang^1^, Junzan Zheng^1^, Qi Wang^1^, Huiwen Li^1^, Hongyang Zhou^1^,** **Zhijun Sun^1^, Hanxi Liu^1^, Jianyi Li^1^, Lin Chen^1^, Qinglin Kang^1^, Zhaoming Qi^1^, Hongwei Jiang^2^,** **Rongsheng Zhu^1^, Xiaoxia Wu^1^, Chunyan Liu^1,*^, Qingshan Chen^1,*^ and Dawei Xin^1,*^**

^1^ Key Laboratory of Soybean Biology of Chinese Ministry of Education, Key Laboratory of Soybean Biology and Breeding/Genetics of Chinese Agriculture Ministry, College of Agriculture, Northeast Agricultural University, Harbin 150030, China.

^2^ Jilin Academy of Agricultural Sciences, Changchun 130033, China

**Author:** Jinhui Wang, PhD, e-mail: [jinhuiwang113@126.com](mailto:jinhuiwang113@126.com);

Jieqi Wang, PhD, e-mail: [jieqi0719@126.com](mailto:jieqi0719@126.com);

Chao Ma, MD, e-mail: mcneau@163.com.

†These authors contributed equally to the work.

***Correspondence:** Chunyan Liu, e-mail: [cyliucn@126.com](mailto:cyliucn@126.com);

Qingshan Chen, e-mail: [qshchen@126.com](mailto:qshchen@126.com);

Dawei Xin, e-mail: [xdawei@163.com](mailto:xdawei@163.com).

**Table S1. Information of Primers Used**

| **No.** | **Name of primer** | **Primer 5’-3’** | **Annotation and Functions** |
| --- | --- | --- | --- |
| 1 | pGWC-*NopD*1.4 | Fwd-AGCAGGAACGGGGGAAATA | Clone the fragment containing *NopD* and putative promoter into pGWC |
|  |  | Rev-GCTCGGTTTTTCTTTTGCG | Clone the fragment containing *NopD* and putative promoter into pGWC |
| 2 | *NopD* Ω*SpeI* | Fwd-GCAATGCCGGCCGCCactagtccgcagccgactcag | Site-directed mutagenesis, mutated *NopD*1.4 had a *Spe1* restriction site |
|  |  | Rev-ctgagtcggctgcggactagtGGCGGCCGGCATTGC | Site-directed mutagenesis |
| 4 | Kan-*SpeI* | Fwd-ACTAGTAGTAAACTGGATGGCTTTCTTG | Clone Kanamycin fragment into pGWC-*NopD*1.4 |
|  |  | Rev-ACTAGTCTTCAGCATCTTTTACTTTCAC | Clone Kanamycin fragment into pGWC-*NopD*1.4 |
| 5 | pJQ200SK-*NopD*Ω | Fwd-TCTAGAAGCAGGAACGGGGGAAATA | Construction of Suicide vector pJQ200SK-*NopD*Ω to generate HH103Ω*NopD* |
|  |  | Rev-CCCGGGGCTCGGTTTTTCTTTTGCG | Construction of Suicide vector pJQ200SK-*NopD*Ω to generate HH103Ω*NopD* |
| 6 | pGWC-*TtsI*1.4 | Fwd-AGGGGTTAGGGCGTTGTTC | Clone the fragment containing *TtsI* and putative promoter into pGWC |
|  |  | Rev-GCGTTGATGCTGTTGGGAG | Clone the fragment containing *TtsI* and putative promoter into pGWC |
| 7 | *TtsI* Ω*SpeI* | Fwd-TGAAATGCGAACGTTactagtggatacggatcttac | Site-directed mutagenesis, mutated *TtsI* 1.4 had a *Spe1* restriction site |
|  |  | Rev-gtaagatccgtatccactagtAACGTTCGCATTTCA | Site-directed mutagenesis |
| 8 | Kan-*SpeI* | Fwd-ACTAGTAGTAAACTGGATGGCTTTCTTG | Clone Kanamycin fragment into pGWC- *TtsI*1.4 |
|  |  | Rev-ACTAGTCTTCAGCATCTTTTACTTTCAC | Clone Kanamycin fragment into pGWC- *TtsI*1.4 |
| 9 | pJQ200SK-*TtsI*Ω | Fwd-GGATCCAGGGGTTAGGGCGTTGTTC | Construction of Suicide vector pJQ200SK- *TtsI*Ω to generate HH103Ω*TtsI* |
|  |  | Rev-GTCGACGCGTTGATGCTGTTGGGAG | Construction of Suicide vector pJQ200SK- *TtsI*Ω to generate HH103Ω*TtsI* |
| 10 | *16S rDNAq* | Fwd-TAAACCACATGCTCCACC | Internal control gene for qQT-PCR |
|  |  | Rev-GATACCCTGGTAGTCCAC | Internal control gene for qQT-PCR |
| 11 | *NopDq* | Fwd-GTTGAATCCGATAGCCTTGTC | qQT-PCR of NopD |
|  |  | Rev-GAATCGCTCCTCTGCAAAT | qQT-PCR of NopD |
| 12 | BARCSOYSSR_19_0531 | Fwd-GAGCGGAACAATCAACCCTA | Validation of consensus QTL by CSSLs |
|  |  | Rev-AAGCAGGTAGAATCTTGAGTTTGA | Validation of consensus QTL by CSSLs |
| 13 | BARCSOYSSR_19_0537 | Fwd-TAAGCTTTGGGGCTGAAAAA | Validation of consensus QTL by CSSLs |
|  |  | Rev-CAAGCATCACACACACCCAT | Validation of consensus QTL by CSSLs |
| 14 | BARCSOYSSR_19_0540 | Fwd-CAATTTTATGCTTCACTCAAACAA | Validation of consensus QTL by CSSLs |
|  |  | Rev-AAAGGAATTCACTTGGTAGCATA | Validation of consensus QTL by CSSLs |
| 15 | BARCSOYSSR_19_0548 | Fwd-ACCACCACCACCTTCTCAAG | Validation of consensus QTL by CSSLs |
|  |  | Rev-AATGCGGTTAAACTTCCCCT | Validation of consensus QTL by CSSLs |
| 16 | BARCSOYSSR_19_0558 | Fwd-AAATTTTGTTGTCCTCCCACA | Validation of consensus QTL by CSSLs |
|  |  | Rev-TCCACCTTTTATTAGTGGGTTCTT | Validation of consensus QTL by CSSLs |
| 17 | BARCSOYSSR_19_0564 | Fwd-CTCCTTTAATTCCCATGCCC | Validation of consensus QTL by CSSLs |
|  |  | Rev-GGCTAGATCATTGGGCTTCA | Validation of consensus QTL by CSSLs |
| 18 | *GmELF1b* | Fwd-GTTGAAAAGCCAGGGGACA | qRT-PCR for validation of *GmELF1b* gene transcription level in Suinong14 |
|  |  | Rev-TCTTACCCCTTGAGCGTGG |  |
| 19 | *Glyma.19G065800* | Fwd-GAATCCTTGACTTGTGCTACTT | qRT-PCR for validation of *Glyma.19G065800* gene transcription level in Suinong14 |
|  |  | Rev-CTCTCTTCTGTCCCCCTTTATT |  |
| 20 | *Glyma.19G066800* | Fwd-TAAGACTGCGTACAACATCCCT | qRT-PCR for validation of *Glyma.19G066800* gene transcription level in Suinong14 |
|  |  | Rev-TGAATAATCTCTAACCGTACAT |  |
| 21 | *Glyma.19G067200* | Fwd-TCCAGAAGCCAAAGGGTCG | qRT-PCR for validation of *Glyma.19G067200* gene transcription level in Suinong14 |
|  |  | Rev-ATACGAGGAGCAGGTTCAT |  |
| 22 | *Glyma.19G068300* | Fwd-AAAACAGAGGTAAGGGGAA | qRT-PCR for validation of *Glyma.19G068300* gene transcription level in Suinong14 |
|  |  | Rev-GGATTCAAAAACCGTCAAA |  |
| 23 | *Glyma.19G068600* | Fwd-TTCTGCTCTGTGGATTTTCATA | qRT-PCR for validation of *Glyma.19G068600* gene transcription level in Suinong14 |
|  |  | Rev-GCATTTATCTCATTTTTTCGTA |  |
| 24 | *Glyma.19G068800* | Fwd-AGAAGTTAGGAGATGCTGTTT | qRT-PCR for validation of *Glyma.19G068800* gene transcription level in Suinong14 |
|  |  | Rev-CTGTGGTTTATTGGAAGTGTG |  |
| 25 | *Glyma.19G069200* | Fwd-CCTTGCTGCTTTGGACTAA | qRT-PCR for validation of *Glyma.19G069200* gene transcription level in Suinong14 |
|  |  | Rev-ATTGGAGGTCTTTTTTATTTTAC |  |

**Table S2. Information of Strains and Vectors**

| **Strain** | **Relevant characteristics** | **Reference** |
| --- | --- | --- |
| ***Escherichia coli*** |  |  |
| DH5α | supE44 lacY169 ( 80lacZM15) hsdR17 recA1 endA1 gyrA96 thi-1 relA1 | Transgene (Transgene Biotech Co., Beijing, China) |
| ***A. tumefaciens*** |  |  |
| EHA105 | C58 (rif^R^) Ti pEHA105 (pTiBo542DT-DNA) (strep^R^) Succinamopine | Transgene (Transgene Biotech Co., Beijing, China) |
| ***Rhizobium* strains** |  |  |
| HH103 | Broad host range bacterium isolated from nodules of Glycine max , Rif^r^ | This work |
| HH103ΩNopD | HH103 insertion mutated containing an Kanamycin resistance gene insertion at position downstream 9bp of start codon of *NopD* nucleotide sequence, Rif^r^, Kan^r^ | This work |
| HH103ΩTtsI | HH103 insertion mutated containing an Kanamycin resistance gene insertion at position downstream 8bp of start codon of *TtsI* nucleotide sequence, Rif^r^, Kan^r^ | This work |
| **Plasmids** |  |  |
| pGWC | Entry clone vector, Cm^r^ | Chen et al. [1] |
| pGWB17 | Binary expression vector, Km^r^ | Nakagawa et al. [2] |
| pJQ200SK | Suicide vector used for directed mutagenesis (Gm^r^) | Quandt and Hynes. [3] |
| pJQ200SK-*NopD*Ω | A 2.4kb Xba1-Sma1 fragment containing *NopD* with a Kanamycin resistance gene inserted into downstream 8bp of start codon of *NopD* the Xba1-SmaI site of pJQ200SK (Gm^r^) | This work |
| pJQ200SK-*TtsI*Ω | A 2.4kb BamH1-Sal1 fragment containing *TtsI* with a Kanamycin resistance gene inserted into downstream 8bp of start codon of *TtsI* the BamH1-SalI site of pJQ200SK (Gm^r^) | This work |
| pRK2013 | Tra^+^ helper plasmid for mobilisation (Kan^r^) | Figurski and Helinski. [4] |

Note: Rifampicin (Rif^r^ ) Kanamycin (Kan^r^) Chloramphenicol (Cm^r^) Gentamicin (Gm^r^)

References cited in Table S2

1. Q. Chen, H. Zhou, J. Chen, X. Wang, Using a modified TA cloning method to create entry clones, Analytical biochemistry. 358.1 (2006), 120-125.
2. T. Nakagawa, T. Kurose, T. Hino, K. Tanakab, M. Kawamukai, Y. Niwa, K. Toyooka, K. Matsuoka, T. Jinbo, T. Kimura, Development of series of gateway binary vectors, pGWBs, for realizing efficient construction of fusion genes for plant transformation, Journal of bioscience and bioengineering. 104.1 (2007), 34-41.
3. J. Quandt, MF. Hynes, Versatile suicide vectors which allow direct selection for gene replacement in gram-negative bacteria, Gene. 127 (1993), 15-21.
4. DH. Figurski, DR. Helinski, Replication of an origin-containing derivative of plasmid RK2 dependent on a plasmid function provided in trans, Proc Natl Acad Sci USA. 76 (1979), 1648-1652.

**Table S3. The soybean germplasms used for nodulation tests.**

| **No.** | **Name** | **Position** |
| --- | --- | --- |
| 1 | Heinong35 (*Glycine max* (L.) Merr.) | Heilongjiang |
| 2 | Qingdou (*Glycine max* (L.) Merr.) | Shanxi |
| 3 | Zheng9525 (*Glycine max* (L.) Merr.) | Henan |
| 4 | Baimaodou (*Glycine max* (L.) Merr.) | Zhejiang |
| 5 | Chidou1 (*Glycine max* (L.) Merr.) | Inner Mongolia |
| 6 | Suinong14 (*Glycine max* (L.) Merr.) | Heilongjiang |
| 7 | ZYD00006 (*G. soja* Sieb. & Zucc*.*) | Heilongjiang |
| 8 | Dongnong594 (*Glycine max* (L.) Merr.) | Heilongjiang |
| 9 | Charleston (*Glycine max* (L.) Merr.) | USA |
| 10 | Kenjian28 (*Glycine max* (L.) Merr.) | Heilongjiang |

**Table S4. Parental and population statistics for nodule traits in the soybean ‘Suinong14’ × ‘ZYD00006’ population.**

|  | **CSSLs (*n* = 142)** | | | | **Parents (average)** | |
| --- | --- | --- | --- | --- | --- | --- |
|  | **Traits** | **Average** | **Standard Deviation** | **Coefficient of Variation** | **Suinong14** | **ZYD00006** |
| HH103 Rif^R^ | Nodule number | 16.8 | 8.7 | 51.78 | 36.3 ± 8.0 | 18.0 ± 4.1** |
|  | Nodule dry-weight (mg) | 10.4 | 7.8 | 75.00 | 57.2 ± 7.1 | 13.0 ± 1.9* |
| HH103 Rif^R^Ω*NopD* | Nodule number | 7.8** | 6.3 | 80.77 | 4.7 ± 2.0** | 8.0 ± 2.1** |
|  | Nodule dry-weight (mg) | 9.6 | 16.6 | 172.91 | 15.5 ± 2.2** | 15.5 ± 2.0 |
| HH103 Rif^R^Ω*TtsI* | Nodule number | 11.7* | 11.7 | 100.00 | 15.3 ± 5.2** | 12.0 ± 3.0** |
|  | Nodule dry-weight (mg) | 11.3 | 12.4 | 109.73 | 20.0 ± 3.9** | 20.0 ± 4.2** |

* indicates significant differences with different inoculations, *p* ≤ 0.05, ** indicates *p* ≤ 0.01.

**Table S5 Annotation of candidate genes associated with NopD**

| **No.** | **Gene** | **Function** |
| --- | --- | --- |
| 1 | *Glyma.19g065800* | Alpha/Beta Hydrolase Fold-Containing Protein |
| 2 | *Glyma.19g066800* | Histone-Lysine N-Methyl Transferase ATX4-Related |
| 3 | *Glyma.19g067200* | Calcium-Binding EF Hand Family Protein |
| 4 | *Glyma.19g068300* | Calmodulin-1-Related |
| 5 | *Glyma.19g068600* | F-Box/LRR-Repeat Protein |
| 6 | *Glyma.19g068800* | lysine-specific demethylase 3 |
| 7 | *Glyma.19g069200* | Protein Phosphatase 2C |

Functional annotation of candidate genes from querying the Phytozome database (<https://phytozome.jgi.doe.gov/pz/portal.html#!info?alias=Org_Gmax>)
